# Protective effect of Rhei Rhizoma on reflux esophagitis in rats via Nrf2-mediated inhibition of NF-κB signaling pathway

**DOI:** 10.1186/s12906-015-0974-z

**Published:** 2016-01-09

**Authors:** O Jun Kwon, Byung Kil Choo, Joo Young Lee, Min Yeong Kim, Sung Ho Shin, Bu-Il Seo, Young-Bae Seo, Man Hee Rhee, Mi-Rae Shin, Gyo-Nam Kim, Chan Hum Park, Seong-Soo Roh

**Affiliations:** 1 Daegyeong Institute for Regional Program Eveluation, Global Bencheodong, Gyeongbuk Techno Park, 300, Sampungdong, Gyeongsan-si, Gyeongsangbuk-do 712-210 Republic of Korea; 2 Department of Crop Agriculture and Life Science, Chonbuk National University, Jeonju, 54896 Republic of Korea; 3 Department of Herbology, College of Korean Medicine, DaeguHaany University, 136, Shinchendong-ro, Suseong-gu Deagu, 42158 Republic of Korea; 4 Laboratory of Veterinary Physiology & Cell Signaling, College of Veterinary Medicine, Kyungpook National University, Daegu, 41566 Republic of Korea; 5 Department of Food Science and Biotechnology, Kyungnam University, 7 Kyungnamdaehak-ro, Masanhappo-gu, Changwon-si 51767 Republic of Korea

**Keywords:** Rhei Rhizoma, Reflux esophagitis, Gastroesophageal reflux disease, Oxidative stress, Inflammation, Antioxidation

## Abstract

**Background:**

Rhei Rhizoma has been widely used as a traditional herbal medicine to treat various inflammatory diseases. The present study was conducted to evaluate its anti-inflammatory activity against experimental reflux-induced esophagitis (RE) in SD rats.

**Methods:**

Rhei Rhizoma was administered at 125 or 250 mg/kg body weight per day for 7 days prior to the induction of reflux esophagitis, and its effect was compared with RE control and normal rats.

**Results:**

Rhei Rhizoma administration markedly ameliorated mucosal damage on histological evaluation. The elevated reactive oxygen species in the esophageal tissue of RE control rats decreased with the administration of Rhei Rhizoma. RE control rats exhibited the down-regulation of antioxidant-related proteins, such as nuclear factor-erythroid 2-related factor 2 (Nrf2) and heme oxygenase-1 (HO-1) expression levels, in the presence of esophagitis; however, the levels with Rhei Rhizoma treatment were significantly higher than those in RE control rats. Moreover, RE control rats exhibited the up-regulation of protein expressions related to oxidative stress in the presence of esophagitis, but Rhei Rhizoma administration significantly reduced the expression of inflammatory proteins through mitogen-activated protein kinase (MAPK)-related signaling pathways. The protein expressions of inflammatory mediators and cytokines by nuclear factor-kappa B (NF-κB) activation were modulated through blocking the phosphorylation of inhibitor of nuclear factor kappa B (IκB)α.

**Conclusion:**

Our findings support the therapeutic evidence for Rhei Rhizoma ameliorating the development of esophagitis *via* regulating inflammation through the activation of the antioxidant pathway.

**Electronic supplementary material:**

The online version of this article (doi:10.1186/s12906-015-0974-z) contains supplementary material, which is available to authorized users.

## Background

Gastroesophageal reflux disease (GERD), including reflux esophagitis, is mainly caused by excessive exposure to the gastric contents, namely acid, pepsin, trypsin, and bile acids, as well as the functional and structural abnormality of the esophagitis [1]. The main symptoms of GERD are acid regurgitation and heartburn [2]. GERD prevalence estimates were 18.1–27.8 % in North America, 8.8–25.9 % in Europe, 2.5–7.8 % in East Asia, 8.7–33.1 % in the Middle East, 11.6 % in Australia, and 23.0 % in South America [3]. The existing therapeutic strategy for GERD is primarily acid suppression such as using antacids, H2-receptor antagonists, and proton pump inhibitors (PPIs) [4]. Despite their well-known efficacy, a number of patients have experienced relapse, incomplete mucosal healing, and the development of severe complications like Barrett’s esophagus. Also, these agents have adverse effects associated with long-term use [5–7].

Recent studies reported that oxidative stress is more important than acid in the pathogenesis of reflux esophagitis in rats [8–10]. Oxidative stress could lead to leukocyte activation, the production of ROS, and increase of tissue damage [11]. In studies carried out on animal models of esophagitis as well as those on human esophageal tissue, ROS were found to be responsible for esophageal tissue damage. ROS, such as superoxide anions (O_2_^−^), hydrogen peroxide (H_2_O_2_), and hydroxyl radicals (∙OH), are released excessively in inflammatory gastroesophageal tissues. Overproduction of ROS can contribute to the immediate development of inflammatory processes. Administration of antioxidants, free radical scavengers, has been reported to prevent esophageal mucosal damage by blocking free radicals [12].

Rhei Rhizoma (rhubarb, Dahuang in Chinese) is one of the traditional herbal medicine widely used in Chinese, Korean, and Japanese pharmacopoeias. It is known to exert several biological effects, which involve purgative, antipyretic, anti-inflammatory, anti-angiogenic, and antineoplastic activities [13–15]. The previous study reported that the administration of rhubarb modulated acute inflammatory response in patients with gastric cancer [16]. Furthermore, we previously reported the increase in free radical scavenging activities of Rhei Rhizoma and Glycyrrhiza Rhizoma combined extract (RGE) in the inflammation induced ROS [17]. Moreover, Wen-Pi-Tan including Rhei Rhizoma exerted potent anti–oxidant effect and anti-lipid peroxidation on oxidative stress and Rhei Rhizoma also promoted incision wound healing and protective effects on gastric ulcer in rats [18, 19]. Primary constituents of Rhei Rhizoma are anthranoids, including sennoside A. Oral intake of Sennoside A is transformed into rhein by bacterial enzymes in the large intestine [20]. Rhein, one of the important active components of Rhei Rhizoma, showed a protective effect against oxidative stress-related endothelial cell injury [21]. The results demonstrated that the antioxidative activity of Rhei Rhizoma can be used therapeutically for inflammation-based GERD including reflux esophagitis. Therefore, we investigated the effects of Rhei Rhizoma on rats with reflux esophagitis to examine its preventive effect against oxidative stress-related inflammation.

## Methods

### Materials

Protease inhibitor mixture solution and ethylenediaminetetraacetic acid (EDTA) were purchased from Wako Pure Chemical Industries, Ltd. (Osaka, Japan). Phenylmethylsulfonyl fluoride (PMSF) was purchased from Sigma Aldrich Co., Ltd. (St. Louis, MO, USA). 2′,7’-Dichlorofluorescein diacetate (DCFH-DA) was obtained from Molecular Probes (Eugene, OR, USA). ECL Western Blotting Detection Reagents and pure nitrocellulose membranes were supplied by GE Healthcare (Piscataway, NJ, USA). Rabbit polyclonal antibodies against Nrf2, HO-1, p-p38, p-ERK1/2, NF-κBp65, and mouse monoclonal antibodies against, and p-IκBα, COX-2, iNOS, TNF-α, IL-6, histone, and β-actin were purchased from Santa Cruz Biotechnology, Inc. (Santa Cruz, CA, USA). Rabbit anti-goat, goat anti-rabbit, and goat anti-mouse immunoglobulin G (IgG) horseradish peroxidase (HRP)-conjugated secondary antibodies were acquired from Santa Cruz Biotechnology, Inc. All other chemicals and reagents were purchased from Sigma Aldrich Co., Ltd. (St. Louis, MO, USA).

### Plant materials

Rhei Rhizoma was purchased from Ominherb Co. (Youngcheon, Korea). A voucher herbarium specimen has been deposited at the Herbarium of DaeguHaany University and was identified by Prof. S.S. Roh, the herbarium leader of this university. Dried slices of Rhei Rhizoma (100 g) were extracted with distilled water (1,000 mL) at room temperature for 2 h, and the solvent was evaporated *in vacuo* to give an extract with a yield of 23.1 %, by weight, of the original Rhei Rhizoma.

### Analysis of Rhei rhizoma by HPLC chromatogram

**Fig. 1:**
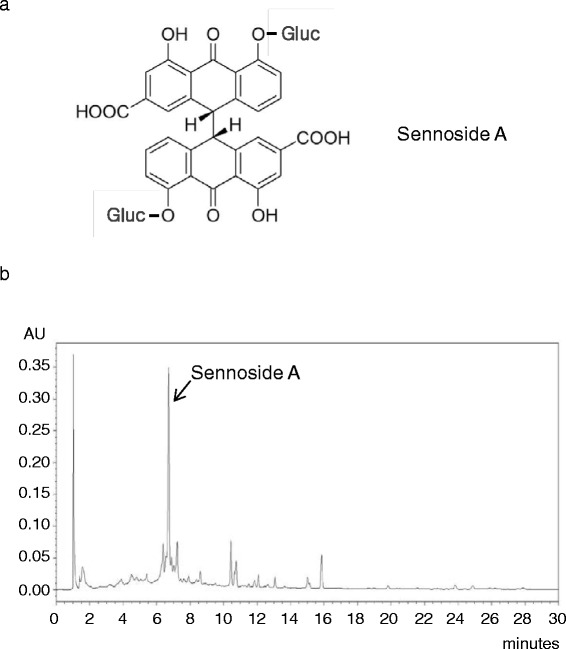
HPLC profile of Rhei Rhizoma at 254 nm wavelength. **a** chemical structure. **b** Sennoside A

The water extract of Rhei rhizoma (1 mg) was dissolved in 1 mL of 50 % methanol with multi-vortexing. We injected 50 μL of the sample into a reverse-phase HPLC using a ZORBAX Eclipse XDB-C18, Analytical 4.6 X 150 mm, 5-μm, with a column temperature of 25 °C. Mobile phase component A = methanol and B = water (10 mM 1-hexanesulfonic acid sodium). The gradient conditions were as follows: 15 % A; 0 min, 50 % A; 15 min, 30 % A, 30 min. The flow rate was 2.0 mL/min. The UV absorbance from 254 nm was monitored using an Agilent 1200 series with an 2998 Photodiode Array Detector from Waters Co. (Manchester, UK). All peaks were assigned by carrying out co-injection tests with authentic samples and comparing them with the UV spectral data. Sennoside A was detected from Rhei rhizoma. The measurement was repeated three times. Representative HPLC result is illustrated in Fig. 1.

### Experimental animals and treatment

Six-week-old male Sprague–Dawley rats (B.W. 180 g - 200 g) were purchased from Samtako (Osan, Korea). Rats were maintained under a 12-h light/dark cycle, housed at a controlled temperature (24 ± 2 °C) and humidity (about 60 %). After adaptation (1 week), the rats (*n* = 24) were divided into four groups, avoiding any inter-group differences in body weight. The normal and vehicle-treated reflux esophagitis groups were given water, while the other groups were orally administered Rhei Rhizoma extract at a dose 125 or 250 mg/kg body weight daily using a stomach tube for 7 consective days (*n* = 6 in each group). The oral doses were determined in a preliminary study, which demonstrated biological activity without toxicity [22]. At the end of the administration period, the rats were fasted for 18 h prior to surgical procedures and kept in raised mesh-bottom cages to prevent coprophagy. And then rats were anaesthetized with an injection of Zoletil at 0.75 mg/kg (Virbac S.A. France). A midline laparotomy was performed to expose the stomach, and then both the pylorus and transitional junction between the forestomach and corpus were first exposed, and later ligated with a 2–0 silk thread but without a plyoric ring, contrary to the procedure originally proposed by Omura et al. [23]. The vagus nerves were left intact. At 6 h after the surgery, all rats were sacrificed. The entire esophagus was removed immediately and examined for gross mucosal injury. The esophageal tissue was immediately frozen in liquid nitrogen and blood samples were collected by vena cava puncture from anesthetized rats. Subsequently, the esophagus and serum were kept at −80 °C until analysis.

### Esophageal lesion ratio

The rat esophagus was cut with scissors in a longitudinal direction from the gastroesophageal junction to the pharynx after sacrifice. The inner mucous was washed away with 0.9 % NaCl and the remaining tissue was laid out on paper. Thereafter, the dissected esophagus was photographed with an optical digital camera (Sony, Tokyo, Japan) and analyzed using the i-solution lite software program. The gross mucosal damage ratio was calculated as follows: the gross mucosal damage ratio (%) = [width of area with esophageal mucosal damage (mm^2^)/width of total area of esophagus (mm^2^)] × 100.

### Histological examination in the esophagus

For microscopic evaluation, the opened esophagus was cut to isolate the middle segment. This segment was fixed in 10 % neutral-buffered formalin and, after embedding in paraffin, cut into 2-μm sections and stained using hematoxylin and eosin (H/E). The stained slices were subsequently observed under an optical microscope and analyzed using the i-Solution Lite software program (Innerview Co. Korea).

### Measurement of gastric pH

After sacrifice, the stomach of each rat was washed with 1 mL of 0.9 % NaCl (pH 7.4) using a 1,000-μL micropipette. The pH of the collected gastric juices were measured using a pH meter (EcoMet, iSTEK Co., Seoul, Korea).

### Measurement of ROS and TBARS levels in the esophagus

Esophageal ROS levels were measured employing the method of Ali et al. [24]. Esophageal tissues were homogenized on ice with 1 mM EDTA-50 mM sodium phosphate buffer (pH 7.4), and then 25 mM DCFH-DA was added to homogenates. After incubation for 30 min, the changes in fluorescence values were determined at an excitation wavelength of 486 nm and emission wavelength of 530 nm. The TBARS level was estimated according to the method of Mihara and Uchiyama [25].

### Preparation of nuclear and post-nuclear fractions

Nuclear protein extraction was performed according to the method of Komatsu [26]. In brief, esophageal tissues were homogenized with ice-cold lysis buffer containing 5 mM Tris–HCl (pH 7.5), 2 mM MgCl_2_, 15 mM CaCl_2_, and 1.5 M sucrose, and then 0.1 M dithiothreitol (DTT) and protease inhibitor mixture solution were added. After centrifugation (10,500 x g for 20 min at 4 °C), the pellet was suspended with extraction buffer containing 20 mM 2-[4-(2-hydroxyethyl)-1-piperazyl] ethanesulfonic acid (pH 7.9), 1.5 mM MgCl_2_, 0.42 M NaCl, 0.2 mM EDTA, and 25 % (v/v) glycerol, and then 0.1 M DTT and protease inhibitor mixture solution were added. The mixture was placed on ice for 30 min, and then the nuclear fraction was prepared by centrifugation at 20,500 × g for 5 min at 4 °C. The post-nuclear fraction was extracted from the esophagus of each rat, as described below. In brief, esophageal tissue was homogenized with ice-cold lysis buffer (pH 7.4) containing 137 mM NaCl, 20 mM Tris–HCl, 1 % Tween 20, 10 % glycerol, 1 mM PMSF, and protease inhibitor mixture solution. The homogenate was then centrifuged at 2,000 × g for 10 min at 4 °C. The protein concentration in each fraction was determined using a Bio-Rad protein kit (Bio-Rad Laboratories, Hercules, CA, USA).

### Immunoblotting analyses

For the estimation of Nrf2, NF-κBp65, and histone, 10 μg of protein from each nuclear fraction was electrophoresed through 8–10 % sodium dodecylsulfate polyacrylamide gel (SDS-PAGE). Separated proteins were transferred to a nitrocellulose membrane, blocked with 5 % (w/v) skim milk solution for 1 h, and then incubated with primary antibodies to Nrf2, NF-κBp65, and histone, respectively, overnight at 4 °C. After the blots were washed, they were incubated with anti-rabbit or anti-mouse IgG HRP-conjugated secondary antibody for 1.5 h at room temperature. Also, 10–15 μg of protein of each post-nuclear fraction of HO-1, p-p38, p-ERK1/2, IκBα, COX-2, iNOS, TNF-α, IL-6, and β-actin was electrophoresed through 8–15 % SDS-PAGE. Each antigen-antibody complex was visualized using ECL Western Blotting Detection Reagents and detected by chemiluminescence with Sensi-Q 2000 Chemidoc (Lugen Sci Co., Ltd., Gyeonggi-do, Korea). Band densities were measured using ATTO Densitograph Software (ATTO Corporation, Tokyo, Japan) and quantified as the ratio to histone or β-actin. The protein levels of groups are expressed relative to those of normal rats (represented as 1).

### Ethical approval

Ethical approval for the study was granted by the University of Daegu Haany Ethical Committee on the 06/02/2015 with certificate number DHU2015-009.

### Statistical analysis

The data are expressed as the mean ± standard deviation. Significance was assessed by one-way analysis of variance (ANOVA) followed by Dunnett’s multiple comparison test (SPSS 11.5.1 for Windows, 2002, SPSS Inc., Chicago, IL, USA). Values of *P* < 0.05 were considered significant.

## Results

### Gross mucosal damage and histological analysis in the esophagus

**Fig. 2:**
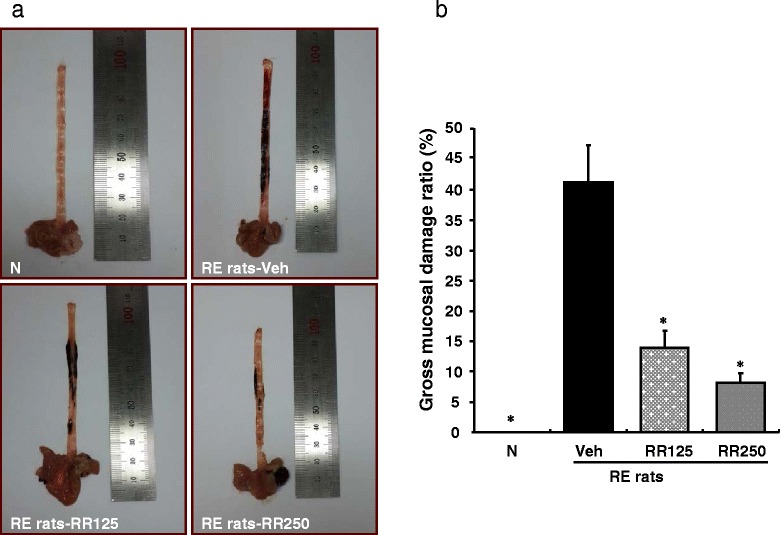
Effects of Rhei Rhizoma on gross esophageal damage. **a** Morphological examination of the esophagus in each group. **b** Gross mucosal damage ratio. N, normal rats; Veh, RE control rats; RR125, Rhei Rhizoma 125 mg/kg-treated reflux esophagitis rats; RR250, Rhei Rhizoma 250 mg/kg mg/kg-treated reflux esophagitis rats. Data are mean ± SD Significance: *P < 0.001 versus RE control rat values

**Fig. 3:**
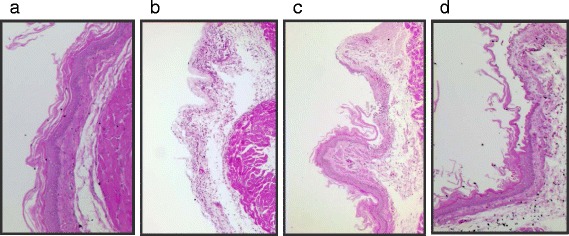
Effect of Rhei Rhizoma on esophageal histology. (original magnification 200×) **a**, normal rats esophagus tissue stained with H&E; **b**, RE control rats esophagus tissue stained with H&E; **c**, Rhei Rhizoma 125 mg/kg-treated reflux esophagitis rats esophagus tissue stained with H&E; **d**, Rhei Rhizoma 250 mg/kg mg/kg-treated reflux esophagitis rats esophagus tissue stained with H&E

Figure 2 shows the results on morphological examination of the esophagus. Morphological changes such as hyperemia and multiple erosions were observed in reflux esophagitis rats. Damage in normal rats was not apparent. The oral administration of Rhei Rhizoma led to a marked decrease of gross mucosal damage (*p* < 0.05). In addition, esophageal tissue stained with H&E revealed no microscopic mucosal changes in normal rat (Fig. 3a). The normal esophagus exhibited a thin epithelial layer with squamous cells and inflammatory cells were not observed in the submucosal layers. By contrast, 6 h subsequent to the induction of RE, RE control rats developed large coalesced longitudinal ulcers in the esophagus sections. Mucosal damage and hyperemia of the epithelial layers and edema and hemorrhage in the mucosa and submucosa were observed in the RE control rats (Fig. 3b). However, the administration of Rhei Rhizoma 125 or 250 mg/kg showed less severe pathological changes (Fig. 3c and d, respectively).

### Gastric pH

**Fig. 4:**
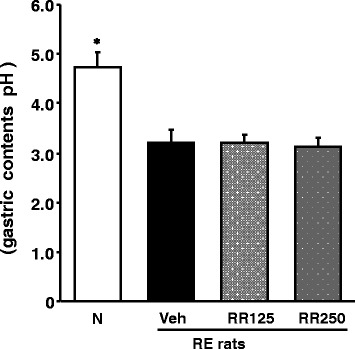
Gastric pH. N, normal rats; Veh, RE control rats; RR125, Rhei Rhizoma 125 mg/kg-treated reflux esophagitis rats; RR250, Rhei Rhizoma 250 mg/kg mg/kg-treated reflux esophagitis rats. Data are mean ± SD. Significance: *P < 0.001 versus RE control rat values

The reflux-induced esophagitis rats displayed a marked decrease in the gastric pH, as shown in Fig. 4. However, the gastric pH was not changed by Rhei Rhizoma administration.

### Biomarkers associated with oxidative stress in the esophagus

As shown in Table 1, the levels of oxidative stress-related biomarkers, ROS and TBARS, in the esophagus of RE control rats were markedly higher than those of normal rats (*p* < 0.01), whereas, by the administration of Rhei Rhizoma at a dose of 250 mg/kg to esophagitis rats, the elevated levels were markedly decreased nearly to the levels of normal rats (*p* < 0.01). The 125 mg/kg Rhei Rhizoma-treated rats also showed a significant decrease (ROS; *p* < 0.05, TBARS; *p* < 0.01).

**Table 1:** Biomarkers associated with oxidative stress in the esophagus

Group	ROS (fluoresence/min/mg protein)	TBARS (nmol/mg protein)
Normal rats	2748 ± 659^**^	7.5 ± 0.4^**^
RE rats		
Veh	29600 ± 8517	14.0 ± 0.8
RR125	9550 ± 5129^*^	8.6 ± 0.1^**^
RR250	3408 ± 661^**^	6.3 ± 0.4^**^

N, normal rats; Veh, RE control rats; RR125, Rhei Rhizoma 125 mg/kg body weight-administrated and RE rats; RR250, Rhei Rhizoma 250 mg/kg body weight-administrated and RE rats. Data are the mean ± SD

Significance: *p < 0.01, **p < 0.001 *vs*. RE control rat values. *n* = 6 in each group

### Oxidative stress-related protein expressions in the esophagus

**Fig. 5:**
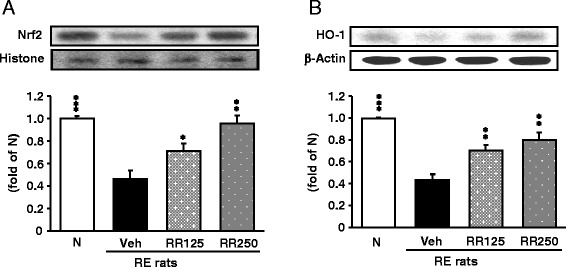
Antioxidant enzyme-related protein expressions in the esophagus. **a** Nrf2 protein expressions. **b** HO-1 protein expressions in each group. N, normal rats; Veh, RE control rats; RR125, Rhei Rhizoma 125 mg/kg-treated reflux esophagitis rats; RR250, Rhei Rhizoma 250 mg/kg mg/kg-treated reflux esophagitis rats. Data are mean ± SD. (*n* = 6) Significance: *P < 0.05, **P < 0.01, ***P < 0.001 versus RE control rat values

Figure 5 showed that esophageal expressions of Nrf2 and HO-1 in RE control rats were significantly decreased compared with those of normal rats (*p* < 0.001). However, Rhei Rhizoma administration adversely regulated the nuclear Nrf2 and cytosolic HO-1 expressions in the esophagus of reflux-induced esophagitis rats. Overall, the ameliorative effects of Rhei Rhizoma 250 mg/kg treatment were superior to those when 125 mg/kg treatment. Especially, our data showed that Rhei Rhizoma stimulated not SOD-1 and catalase but Nrf2 pathway. (Additional file 1: Figure S1)

### MAPK-related protein expressions in the esophagus

**Fig. 6:**
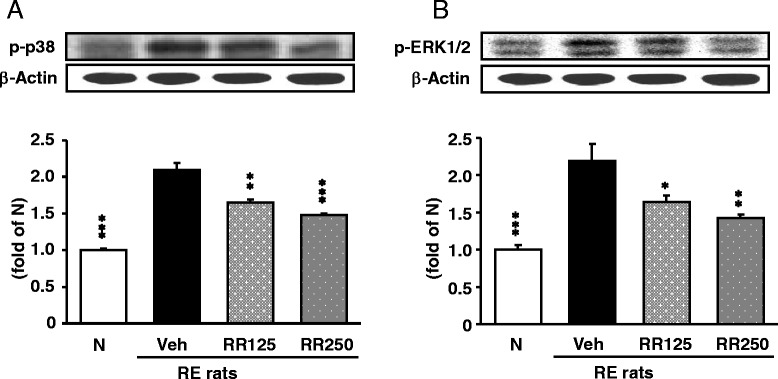
MAPK-related protein expressions in the esophagus. **a** p-p38 protein expressions. **b** p-ERK1/2 protein expressions in each group. N, normal rats; Veh, RE control rats; RR125, Rhei Rhizoma 125 mg/kg-treated reflux esophagitis rats; RR250, Rhei Rhizoma 250 mg/kg mg/kg-treated reflux esophagitis rats. Data are mean ± SD. (*n* = 6) Significance: *P < 0.05, **P < 0.01, ***P < 0.001 versus RE control rat values

MAPK-related protein expressions were augmented in the esophagus of RE control rats compared to the normal rats (*p* < 0.001), but the oral administration of Rhei Rhizoma significantly decreased the expressions of p-p38 and p-ERK1/2 in a dose-dependent manner, as shown in Fig. 6.

### Inflammation-related protein expressions in the esophagus

**Fig. 7:**
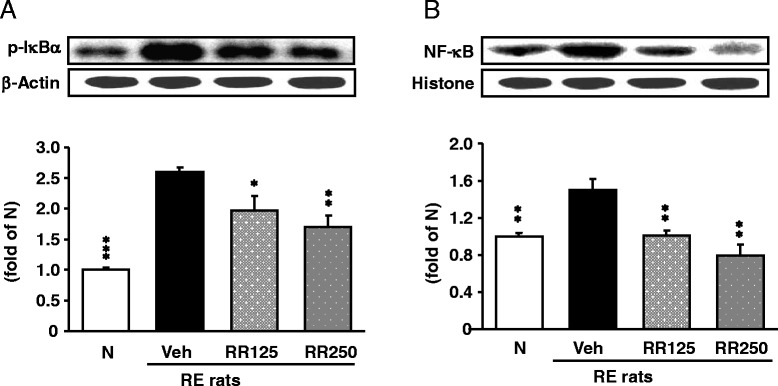
p-IκBα and NF-κBp65 protein expressions in the esophagus. **a** p-IκBα protein expressions. **b** NF-κBp65 protein expressions in each group. N, normal rats; Veh, RE control rats; RR125, Rhei Rhizoma 125 mg/kg-treated reflux esophagitis rats; RR250, Rhei Rhizoma 250 mg/kg mg/kg-treated reflux esophagitis rats. Data are mean ± SD. (*n* = 6) Significance: *P < 0.05, **P < 0.01, ***P < 0.001 versus RE control rat values

**Fig. 8:**
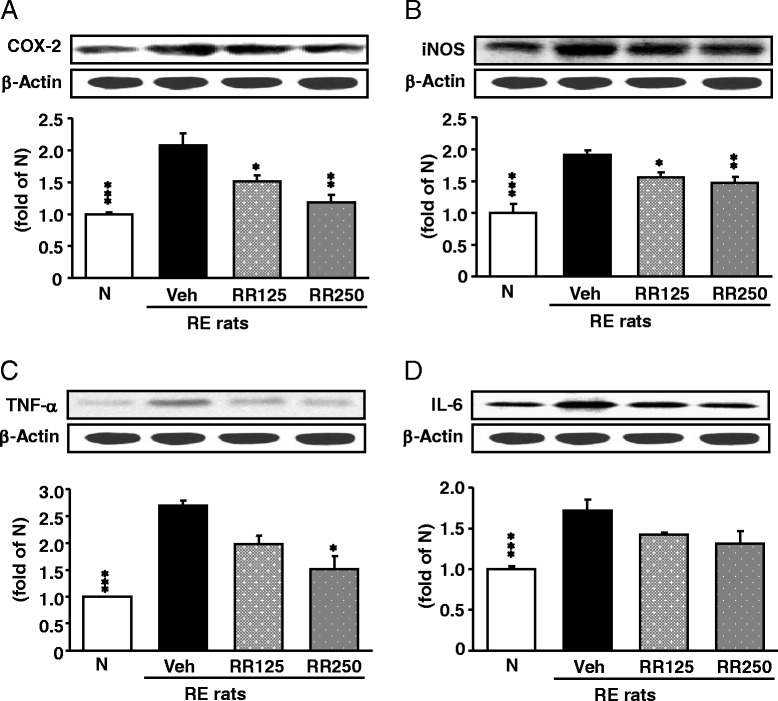
COX-2, iNOS, TNF-α, and IL-6 protein expressions in the esophagus. **a** COX-2 protein expressions. **b** iNOS protein expressions **c** TNF-α protein expressions **d** IL-6 protein expressions in each group. N, normal rats; Veh, RE control rats; RR125, Rhei Rhizoma 125 mg/kg-treated reflux esophagitis rats; RR250, Rhei Rhizoma 250 mg/kg mg/kg-treated reflux esophagitis rats. Data are mean ± SD. (*n* = 6) Significance: *P < 0.05, **P < 0.01, ***P < 0.001 versus RE control rat values

As shown in Fig. 7, protein levels such as p-IκBα (*p* < 0.001) and NF-κBp65 (*p* < 0.01) were enhanced in the esophagus of RE control rats, whereas these elevated levels were significantly reduced in Rhei Rhizoma-treated RE rats dose-dependently. Especially, the NF-κBp65 level was lowered nearly to that of normal rats by 250 mg/kg Rhei Rhizoma treatment (*p* < 0.01). Moreover, the expression levels of COX-2, iNOS, TNF-α were also enhanced in the esophagus of RE control rats in a dose-dependent manner by Rhei Rhizoma treatment (Fig. 8). Rhei Rhizoma 250 mg/kg treatment effectively suppressed more than 125 mg/kg treatment. However, IL-6 showed a tendency to decrease without significance.

## Discussion

In present study, we identified a novel function of Rhei Rhizoma as a effective therapeutic modulator for Reflux Esophagiti (RE) and elucidated the tagrget mechanisms. The administration of Rhei Rhizoma significantly reduced esophageal mucosal damage and and the levels of RE related pathogenic inflammatory mediators and cytokines. RE, commonly referred as GERD, is a chronic and relapsing esophageal pathology, having a significant impact on the quality of life and healthcare costs [27]. Gastroesophageal reflux has also been implicated in the development of esophageal stricture and Barret’s esophagus, and was associated with a greater risk of the development of esophageal cancer. The etiology of reflux esophagitis is complex and multifactorial rather than a single cause, including hypersensitivity of the esophageal mucosal to physiological reflux, reduced mucosal defense mechanisms, and gastric motility disturbances [28]. It is generally known that reflux of the gastric contents causes inflammation, ulceration, and destruction of the normal squamous epithelium of the esophagus [8]. In recent studies, it was shown that mucosal damage in reflux esophagitis is mediated primarily by oxygen-derived free radicals. The administration of various free radical scavengers has been reported to prevent esophageal mucosal damage [29].

The Rhei Rhizoma has richly sennoside A which was included in dianthrone glycosides. Sennoside A is transformed into an bioactive metabolite, rheinanthrone, and then rhein by intestinal bacteria. Particularly Sennoside A exerts the gastroprotective activity via the up-regulation of prostaglandin E2 and the Inhibition of H(+)/K(+)-ATPase [30]. Rhein induces apoptosis of gastric cancer cells through an intrinsic mitochondrial pathway [31]. The derivatives of Sennoside A has been reported to show effective antioxidative activities against H_2_O_2_, light, and γ-radiation induced oxidative stress *via* the suppression of ROS production and scavenging of free radicals [32, 33]. However, the mechanisms underlying the effects of Rhei Rhizoma have yet to be investigated in an experimental model of reflux esophagitis. Therefore, the present study was conducted using an experimental reflux esophagitis model.

The general pathophysiology of gastric disorders is an imbalance between digestive and protective factors in the stomach, such as acid-pepsin secretion, the mucosal barrier, mucus secretion, blood flow, cellular regeneration, prostaglandins, and epidermal growth factors. The pylorus ligation model shows increases in the gastric volume, acid-pepsin concentration, and acid-pepsin output [34]. These stresses have been reported to induce gastric ulcers and increase free radical generation aside from acid-pepsin factors. In this study, RE control rats showed a markedly decreased gastric pH, similarly to another study, and elevated oxidative stress-related factors. However, the administration of Rhei Rhizoma did not affect regulation of the gastric pH. Nevertheless, the esophageal macroscopic and histological lesions were reduced markedly through the different mechanism without regulating the gastric pH [35].

ROS were reported to play a role in the pathogenesis of several gastrointestinal diseases such as inflammatory bowel disease and peptic ulcer [9]. ROS generated in the process of reflux esophagitis were found to be responsible for esophageal tissue damage [36], and this finding was further supported by studies showing that tissue damage could be prevented by the antioxidant activity. ROS induces alterations in the Nrf2 complex, and its gene transcription, such as that of HO-1, is enhanced. Nrf2, which is a redox-sensitive transcription factor, plays a vital role in protection against oxidant-induced cellular injury. Therefore, the Nrf2/HO-1 pathway could be a biomarker of oxidative stress and an adaptive response under pathological conditions [37]. In the present study, esophageal reflux induced esophageal tissue damage triggered by an ROS-sensitive pathway, and oxidative stress was reduced significantly by the administration of Rhei Rhizoma. Moreover, reflux esophagitis rats showed decreased expressions of Nrf2 and HO-1 in esophageal tissues compared with normal rats; however, Rhei Rhizoma administration effectively alleviated oxidative stress and resulted in the up-regulation of Nrf2 and HO-1. Meanwhile, Rhei Rhizoma showed a tendency to increase the SOD and catalase levels without significance. (see Additional file 1: Figure S1).

The accumulation of ROS in gastric epithelium has been linked to gastric carcinogenesis (as well as inflammation). ROS overexpression activates MAPK including p38 and ERK1/2. The MAPK cascades on p38 and ERK are proving to play major roles in the regulation of intracellular metabolism and gene expression in many areas, including disease, apoptosis, and cellular responses to external stresses. Furthermore, the phosphorylation of p38 and ERK1/2 MAPK leads to NF-κB translocation. The NF-κB element is believed to be the main regulator of the inducible expression of inflammatory genes. Activated ERK1/2 induces the dissociaton of IκBα to NF-κB, allowing the nuclear translocation and DNA-binding of NF-κB, and p38 induces the expression of p65 and p50 [38, 39]. In this study, increased expressions of p-ERK1/2 and p-p38 were observed in esophageal tissues of RE control rats, and were decreased by the administration of Rhei Rhizoma. The results of the present study show that Rhei Rhizoma blocked the phosphorylation of IκBα and prevented the nuclear translocation NF-κB in esophageal tissue. Namely, the administration of Rhei Rhizoma markedly suppressed NF-κB activation through the direct inhibition of phosphorylation of IκBα

Excessive activation of NF-κB can lead to serious inflammatory diseases and cancers. NF-κB promotes the transcription of target genes such as TNF-α and IL-6, and regulates the expression of inducible enzymes such as COX-2 and iNOS. When monocytes and macrophages are exposed to inflammatory stimuli, they secrete cytokines such as TNF-α and IL-6. TNF-α induces a number of physiological effects, including septic shock, inflammation, and cytotoxicity [40]. IL-6 is a pleiotropic cytokine, and has both proinflammatory and anti-inflammatory functions that affect processes ranging from immunity to tissue repair and metabolism. It promotes the differentiation of B cells into plasma cells, activates cytotoxic T cells, and regulates bone homeostasis [41]. The role of COX-2 was augmented under inflammatory conditions, such as reflux esophagitis and Barrett’s esophagus [42], and iNOS, which generates NO, is an important mediator of reflux-induced cell signaling in esophageal cells [43]. The up-regulation of iNOS expression results in the overproduction of NO. NO reacts with O_2_^−^ and forms ONOO^−^. ONOO^−^ can directly cause DNA damage and participate in inflammation-related carcinogenesis [44]. In the present study, Rhei Rhizoma treatment of the reflux esophagitis model significantly decreased esophageal protein up-regulation of NF-κB-related inflammatory mediators (COX-2 and iNOS) (Fig. 8). In addition, the elevated protein expressions of TNF-α and IL-6 were significantly down-regulated by the administration of Rhei Rhizoma (Fig. 8).

**Fig. 9:**
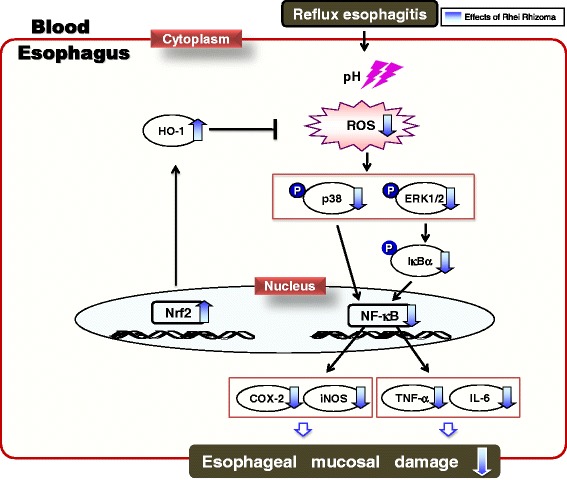
Possible mechanism of Rhei Rhizoma in the esophagus of reflux-induced esophagitis rats

A recent study showed that ROS are one of the most important factors in the pathogenesis of esophageal mucosal injury mediated by oxidative stress in an experimental model of reflux esophagitis. In the present study, the administration of Rhei Rhizoma reduced the oxidative stress biomarker without modifying the gastric pH. These results revealed an antioxidant effect through Nrf2-mediated HO-1 induction. Furthermore, the anti-inflammatory effect of Rhei Rhizoma suggested that the inactivation of NF-κB by blocking MAPK such as through p38 and ERK1/2 signaling pathways leads to an inhibition of the release of proinflammatory cytokines and mediators. That is, Rhei Rhizoma ameliorated inflammation with esophageal mucosal injury caused by experimental reflux esophagitis in rats, as shown in Fig. 9.

## Conclusions

The administration of Rhei Rhizoma effectively ameliorates inflammatory damage in the esophageal mucosa through activation of the Nrf2/HO-1 antioxidant pathway.

## Additional file

Additional file 1: Figure S1. Antioxidant enzyme-related protein expressions in the esophagus. **a** SOD-1 protein expressions. **b** catalase protein expressions in each group. N, normal rats; Veh, RE control rats; RR125, Rhei Rhizoma 125 mg/kg-treated reflux esophagitis rats; RR250, Rhei Rhizoma 250 mg/kg treated reflux esophagitis rats. Data are mean ± SD (*n* = 6). The esophageal expressions of SOD-1 and catalase in RE control rats showed a tendency to decrease without significance compared with those of normal rats. The decreased SOD-1 and catalase levels slightly increased by the treatment of Rhei Rhizoma. However, it did not exist a significance among the experimental group. (PPT 172 kb)

## Abbreviations

GERD: Gastroesophageal reflux disease
ROS: Reactive oxygen species
TBARS: 2-thiobarbituric acid-reactive substance
Nrf2: Nuclear factor-erythroid 2-related factor 2
HO-1: Heme oxygenase-1
p-IκBα: Phosphor-inhibitor of nuclear factor kappa Bα
NF-κB p65: Nuclear factor-kappa B p65
MAPK: Mitogen-activated protein kinase
p-ERK1/2: phosphor-extracellular signal-regulated kinase 1/2
p-p38: phosphor-p38
COX-2: Cyclooxygenase-2
iNOS: Inducible nitric oxide synthase
TNF-α: Tumor necrosis factor-alpha
IL-6: Interleukin-6
NO: Nitric oxide
ONOO^−^: Peroxynitrite
