# Supplementary material for: Protective effect of Rhei Rhizoma on reflux esophagitis in rats via Nrf2-mediated inhibition of NF-κB signaling pathway
Source: BMC Complement Altern Med. 2016 Jan 9;16:7. doi: 10.1186/s12906-015-0974-z (PMC4707002; doi:10.1186/s12906-015-0974-z)
Supplement: Additional file 1: Figure S1. — Antioxidant enzyme-related protein expressions in the esophagus. a SOD-1 protein expressions. b catalase protein expressions in each group. N, normal rats; Veh, RE control rats; RR125, Rhei Rhizoma 125 mg/kg-treated reflux esophagitis rats; RR250, Rhei Rhizoma 250 mg/kg treated reflux esophagitis rats. Data are mean ± SD (n = 6). The esophageal expressions of SOD-1 and catalase in RE control rats showed a tendency to decrease without significance compared with those of normal rats. The decreased SOD-1 and catalase levels slightly increased by the treatment of Rhei Rhizoma. However, it did not exist a significance among the experimental group. (PPT 172 kb) [file 12906_2015_974_MOESM1_ESM.ppt]

## Slide 1
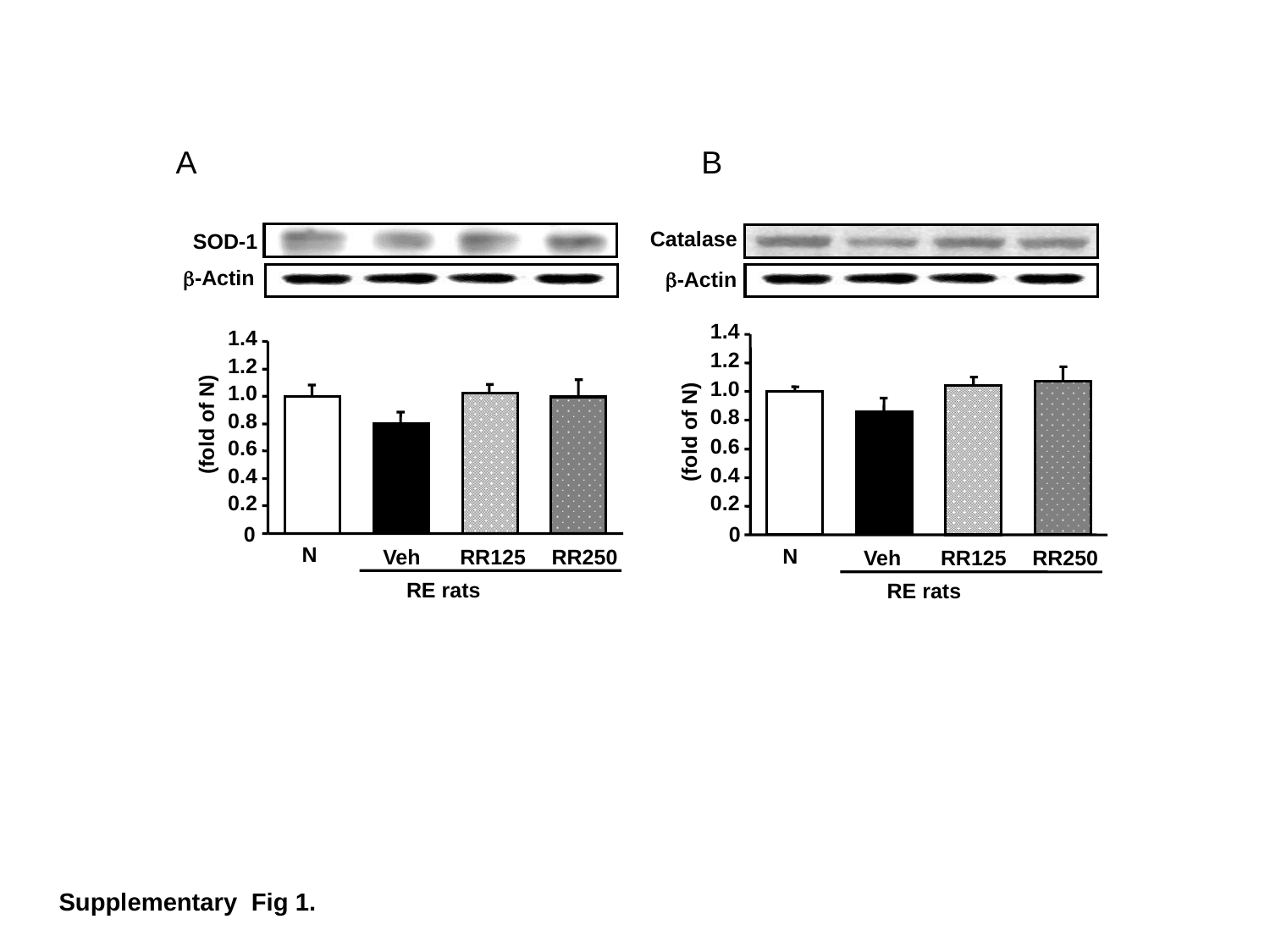

A
B
Catalase
SOD-1
β-Actin
β-Actin
1.4
1.4
1.2
1.2
1.0
1.0
0.8
0.8
(fold of N)
(fold of N)
0.6
0.6
0.4
0.4
0.2
0.2
0
0
N
N
Veh
RR125
RR250
Veh
RR125
RR250
RE rats
RE rats
Supplementary Fig 1.
